# The Practical Medicine Series of Year Books

**Published:** 1903-05

**Authors:** 


### BOOK REVIEWS.

---

The Practical Medicine Series of Year Books. Volume III. The Eye, Ear, Nose and Throat. For 1902. Edited by Casey A. Wood, M.D., Albert H. Andrews, M.D., T. Melville Hardie, M.D.

This little work of 310 pages has again appeared along with nine other volumes, embracing the whole domain of medicine and surgery. In this volume all the notable advances in the domain of the eye, ear, nose and throat have been chronicled and compiled in a manner for ready reference, making it a work of distinct value. The editors are men eminently qualified for this work, and they have put in good shape just the matter which is worth knowing and preserving. We consider such a work as this to be of distinct value to men who desire to keep abreast of the times, and who do not wish to be burdened with innumerable monthly periodicals, which are not only expensive but time-taking in their perusal. The volume is neat and attractive and the paper good. In this respect it is somewhat of an improvement over the last year's volume. A work of this kind should certainly find a ready sale. R.

